# In Situ Observation of the Tensile Deformation and Fracture Behavior of Ti–5Al–5Mo–5V–1Cr–1Fe Alloy with Different Microstructures

**DOI:** 10.3390/ma14195794

**Published:** 2021-10-03

**Authors:** Suping Pan, Mingzhu Fu, Huiqun Liu, Yuqiang Chen, Danqing Yi

**Affiliations:** 1 School of Materials Science and Engineering, Central South University, Changsha 410083, China; pan-su-ping@163.com (S.P.); fumingzhu@csu.edu.cn (M.F.); yioffice@csu.edu.cn (D.Y.); 2 Advanced Research Center, Central South University, Changsha 410083, China; 3 State Key Laboratory of Powder Metallurgy, Central South University, Changsha 410083, China; 4 Hunan Engineering Research Center of Forming Technology and Damage Resistance Evaluation for High Efficiency Light Alloy Components, Hunan University of Science and Technology, Xiangtan 411201, China

**Keywords:** Ti–5Al–5Mo–5V–1Cr–1Fe alloy, in situ observation, slip band, microcrack, fracture mechanism

## Abstract

The plastic deformation processes and fracture behavior of a Ti–5Al–5Mo–5V–1Cr–1Fe alloy with bimodal and lamellar microstructures were studied by room-temperature tensile tests with in situ scanning electron microscopy (SEM) observations. The results indicate that a bimodal microstructure has a lower strength but higher ductility than a lamellar microstructure. For the bimodal microstructure, parallel, deep slip bands (SBs) are first noticed in the primary α (α_p_) phase lying at an angle of about 45° to the direction of the applied tension, while they are first observed in the coarse lath α (α_L_) phase or its interface at grain boundaries (GBs) for the lamellar microstructure. The β matrix undergoes larger plastic deformation than the α_L_ phase in the bimodal microstructure before fracture. Microcracks are prone to nucleate at the α_p_/β interface and interconnect, finally causing the fracture of the bimodal microstructure. The plastic deformation is mainly restricted to within the coarse α_L_ phase at GBs, which promotes the formation of microcracks and the intergranular fracture of the lamellar microstructure.

## 1. Introduction

Due to their strength, corrosion resistance, and heat resistance, near-β-titanium alloys have been widely used in aerospace and automotive engineering as structural materials [1]. With the continuous progress of aerospace and automotive technology, more challenging requirements are imposed on the mechanical properties of titanium alloys [2]. The design of titanium alloys with better mechanical properties is therefore the focus of many studies [3,4,5,6].

Previous studies found that the mechanical properties of titanium alloys are closely related to the morphology and distribution of the α phase [7,8,9]. Two types of typical microstructure (bimodal and lamellar microstructures) can be obtained in titanium alloys by different heat treatments [10]. Many studies concerning the microstructure–property relationship of titanium alloys showed that bimodal variants have a higher ductility and lower strength than their lamellar counterparts [11,12,13]. Qin et al. [14] found that the yield strength (σ_0.2_) of lamellar variants reaches 1900 MPa while that of bimodal alloys is around 1600 MPa. Zheng et al. [15] found that the elongation of bimodal variants is 11.5–14.5% and that of lamellar alloys is 4.5–9.5%. Therefore, the microstructure plays an essential role in determining the mechanical properties of titanium alloys.

Many studies have been performed to reveal the essential mechanism underpinning the different mechanical properties of titanium alloys induced by bimodal and lamellar microstructures. Wu et al. [16] found that the higher ductility of bimodal alloys can be mainly attributed to the more numerous deformation mechanisms available to the equiaxed primary α phase (α_p_) in bimodal alloys (including dislocation slips, twins, and shear bands) than those of the α lath (α_L_) in lamellar structures. Some believed that the tensile fracture of near-β-titanium alloys is sensitive to micro-void nucleation. Qin et al. [17] found that the tensile fracture of lamellar alloys is a process of the initiation of nano-scale voids, followed by their growth and coalescence in the deformation band, and transgranular shearing. The reason for the formation of micro-voids is the stress concentration caused by the difference in the strength of the α phase and β matrix. Stress concentration at the GBs is derived from the precipitation of the α_L_ phase along the α/β interface, which results in micro-void nucleation. This behavior is mainly responsible for the low ductility of lamellar alloys. Prior research into the deformation and properties of materials is based on ex situ test methods, which make it difficult to provide direct evidence of the deformation and fracture behavior; therefore, detailed information concerning the evolution of a microstructure, and its corresponding effect on the fracture process under tension, remains unclear.

Recently, in situ tensile SEM observation has become a powerful and effective tool to evaluate the deformation behavior from a microstructural perspective by capturing the microstructural evolution dynamically [18,19]. Huang et al. [20] indicated that localized stress concentration at the GBs derived from the geometric incompatibility between neighboring α grains was mainly responsible for microcrack formation in a Ti–6Al alloy. Zhang et al. [21] studied the deformation mechanism of a Ti–5Al–2Sn–2Zr–4Cr–4Mo alloy with a bimodal microstructure, implying that the α_p_ had high compatibility of deformation and the slip line in the α_p_ phase was the primary deformation mechanism. Shao et al. [22] found that microcracks were primarily initiated along the α_L_ phase at the edges of the sample. To date, however, the essential mechanism of the effect of microstructure on the mechanical properties of titanium alloys remains unclear, which hinders any further attempts to improve the mechanical properties of alloys.

In this study, Ti–5Al–5Mo–5V–1Cr–1Fe alloy samples with bimodal and lamellar microstructures were prepared through heat treatments. Their room-temperature tensile deformation process and fracture behavior were monitored in real time by in situ SEM observations. On this basis, the aim of this work is to ascertain the essential effects of microstructure on the mechanism of tensile deformation and fracture of titanium alloys. The results of this work are expected to provide a basis for future improvement in the mechanical properties of such titanium alloys.

## 2. Materials and Methods

### 2.1. Materials

The as-received material in this study was a forged Ti–5Al–5Mo–5V–1Cr–1Fe alloy provided by Baoti Group Ltd (Baoji, China) as cuboidal specimens (98.0 mm in length, 20.0 mm in width, and 7.0 mm in height) with a nominal composition of 5.07% Al, 4.81% Mo, 4.74% V, 1.06% Fe, 0.95% Cr, and the rest Ti (all in wt%). The α + β/β transition temperature (*T_α + β__→__β_*) of this alloy is about 865 °C.

**Figure 1:**
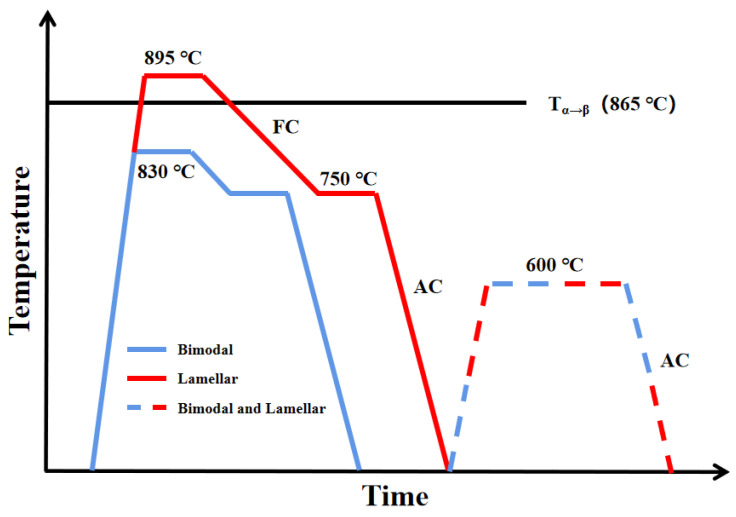
Heat treatment routes for bimodal and lamellar samples.

To obtain the desired microstructure, two as-received cuboids were, respectively, solution-treated at 830 °C (below *T_α + β__→__β_*) and 895 °C (above *T_α + β__→__β_*) for 2 h. Then, these cuboids were cooled in a furnace (FC) to 750 °C and held for 2 h before air cooling (AC). Thereafter, they were aged at 600 °C for 8 h, before air cooling (AC) to room temperature (Figure 1).

### 2.2. In Situ Tensile Test

**Figure 2:**
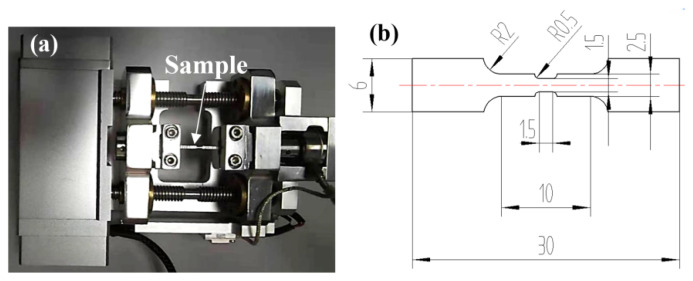
(**a**) Mechanical test bench; (**b**) geometry and dimensions of in situ tensile test specimens at room temperature (units: mm).

The sample for the in situ tensile test was cut by wire-electrode cutting with a gauge length of 1.5 mm, a gauge width of 1.5 mm, and a thickness of 1.0 mm (Figure 2). It was mechanically polished using emery papers with SiC (5 μm, 3 µm, 1 µm, and 0.25 µm). Then, it was chemo-mechanically polished using Al_2_O_3_ (0.04 µm) suspension to remove the work-hardening resulting from previous mechanical polishing. A micro-stage (Figure 2a) and Mini MTS (Liweiauto Ltd., Hangzhou, China) controller (with a maximum load capacity of 2500 N) were employed to clamp the sample and control the tensile strain rate, respectively. The Mini MTS system was adopted to measure the mechanical properties of in situ samples. Before in situ testing, the sample was mechanically polished and then stretched at a speed of 1.5 μm/s at room temperature in vacuum.

Processes during the in situ tensile deformation in bimodal and lamellar microstructures were observed using a JSM-5600 field-emission gun SEM (JEOL Ltd., Tokyo, Japan) with an accelerating voltage of 15 kV. Several interruptions were allowed by the loading system during tensile testing: this allowed the load to be held while capturing the SEM micrographs, after which the tensile test was resumed from the same applied load and displacement at the same rate. Three repeated in situ tensile tests were carried out under each set of experimental conditions, and typical results were provided. The tensile direction for the corresponding SEM images printed herein was parallel to the vertical direction.

Electron backscattered diffraction (EBSD) measurements were conducted before and after in situ tensile tests using an AZtec system (Oxford Instruments Group, Oxford, UK) coupled to a Hitachi-Regulus 8230 cold field emission SEM (Hitachi High-Technologies Corporation, Tokyo, Japan). The operating voltage used was 20 kV to optimize the quality of the diffraction patterns. The EBSD samples were electropolished using a solution of 8% perchloric acid (HClO_4_) and 92% CH_4_O at −25 °C.

## 3. Results

### 3.1. Microstructure of As-Heat-Treated Samples

**Figure 3:**
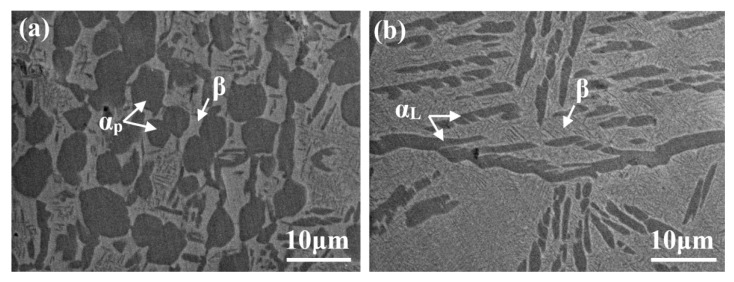
BSE-SEM images of samples before stretching in situ; (**a**) bimodal microstructure; (**b**) lamellar microstructure.

Figure 3a shows backscattered electron (BSE)-SEM images of bimodal samples before in situ tensile testing. Since this sample was solution-treated at a temperature below *T_α + β__→__β_*, some coarse globular α_p_ phases were maintained, which were evenly distributed in the β matrix. For the lamellar microstructure seen in Figure 3b, α_p_ was completely dissolved during solution treatment at a temperature above *T_α + β__→__β_* (i.e., 895 °C for 2 h), while coarse α_L_, formed during subsequent low-temperature aging treatment, was distributed along GBs of the β matrix.

### 3.2. The Stress–Displacement Curves during In Situ Testing

**Figure 4:**
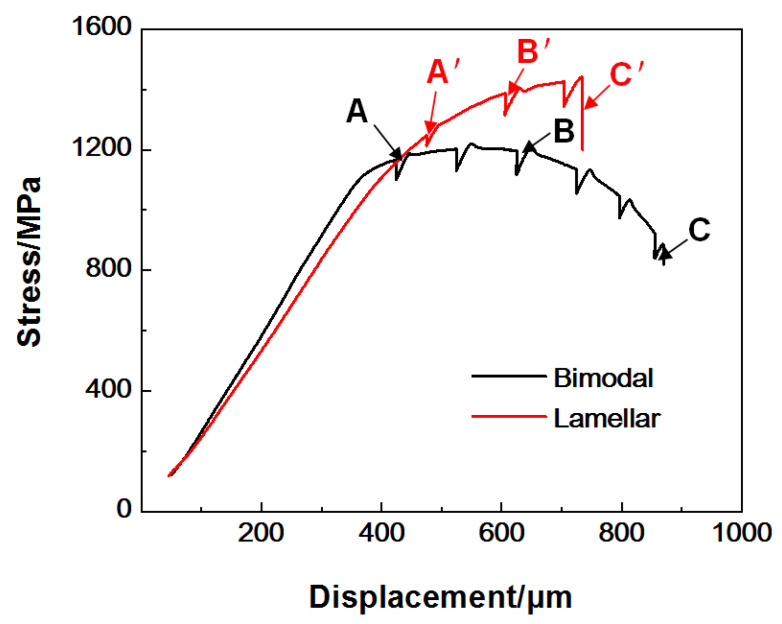
The stress–displacement curves of samples with bimodal and lamellar microstructures stretched in situ at room temperature.

The stress–displacement curves of bimodal and lamellar microstructures under in situ stretching are demonstrated in Figure 4. The drops in the curves are caused by slight stress relaxation during the pauses for SEM imaging, in which three typical drops for each sample are marked, respectively (A, B, and C for the bimodal microstructure and A’, B’, and C’ for the lamellar microstructure).

As can be seen from Figure 4, σ_0.2_ and the ultimate tensile stress (σ_th_) of the bimodal microstructure are 1041.8 and 1220.5 MPa, which are much lower than those of specimens with a lamellar microstructure (σ_0.2_ = 1102.7 MPa, σ_th_ = 1441.3 MPa). The maximum tensile displacement of the bimodal microstructure is about 872 μm, while that of the lamellar microstructure is about 736 μm. This result indicates that the lamellar microstructure is stronger, but less ductile than the bimodal microstructure, which is consistent with findings from previous studies [11,12,13,14,15].

### 3.3. Microstructure Evolution during In Situ Stretching

#### 3.3.1. Microstructure Evolution of Bimodal Microstructure

**Figure 5:**
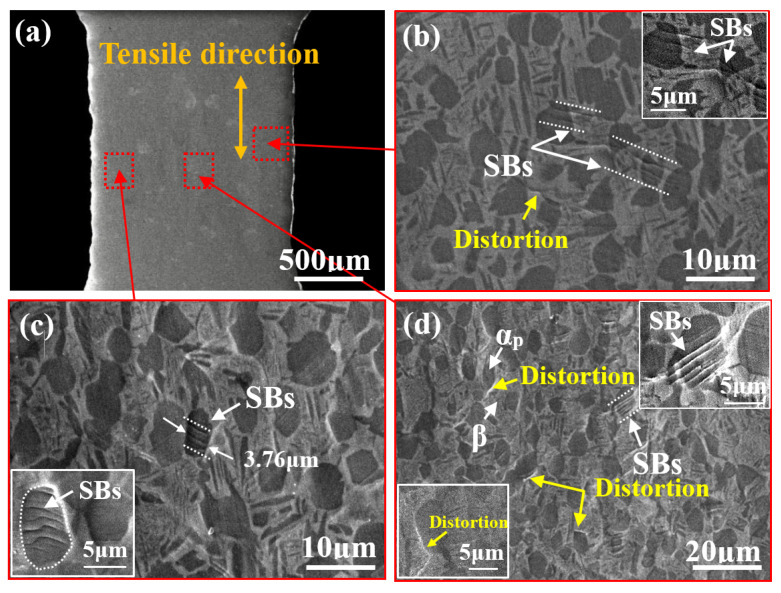
(**a**) In situ SEM images of the bimodal microstructure at position A; (**b**) magnified image showing SBs passing through the α_p_/β interface; (**c**) SBs formed in the region containing the α_p_ phase; (**d**) distortion at the α_p_/β interface region.

Figure 5a illustrates the in situ SEM images of the bimodal microstructure at position A. At this stage, some parallel and deep SBs can be seen in a small number of α_p_ phase regions. These SBs generally lie at an angle of 41° to 49° with the tensile direction. As shown in Figure 5c, most of them are quite short (only several microns) and strictly confined within a single α_p_ phase, while some of them not only cross the whole α_p_ phase grain, but also pass through the α_p_/β interface and enter the region containing the β matrix (Figure 5b). In addition, there is a certain region of distortion arising at the α_p_/β interface (Figure 5d), which may be attributed to the deformation incompatibility between α_p_ and β due to their different crystal structures.

**Figure 6:**
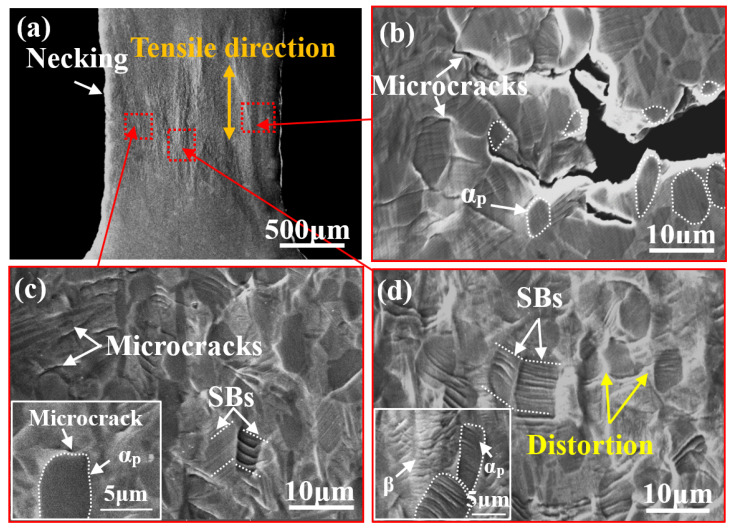
(**a**) In situ SEM images of the bimodal microstructure at position B; (**b**) magnified image showing microcracks formed on the edge of the sample; (**c**) microcracks initiated from the α_p_/β interface; (**d**) distortion was aggravated around the α_p_/β interface.

Figure 6 illustrates BSE-SEM images of bimodal samples stretched at position B. Obvious necking appears (Figure 6a) and some microcracks form on the edge of the sample (Figure 6b) caused by the increasing strain. The microcrack tends to propagate along the α_p_/β interface and gradually grows to the center of the sample, as shown in Figure 6b. Noticeable SBs can be found in the β matrix adjacent to regions of α_p_ phase and the distortion at the α_p_/β interface increases in severity (Figure 6d). This indicates that a higher stress concentration arises at this region near the α_p_/β interface. In addition, Figure 6c shows that the SBs in the α_p_ phase become deepened and some tiny microcracks are also initiated in the β matrix close to regions containing the α_p_ phase.

**Figure 7:**
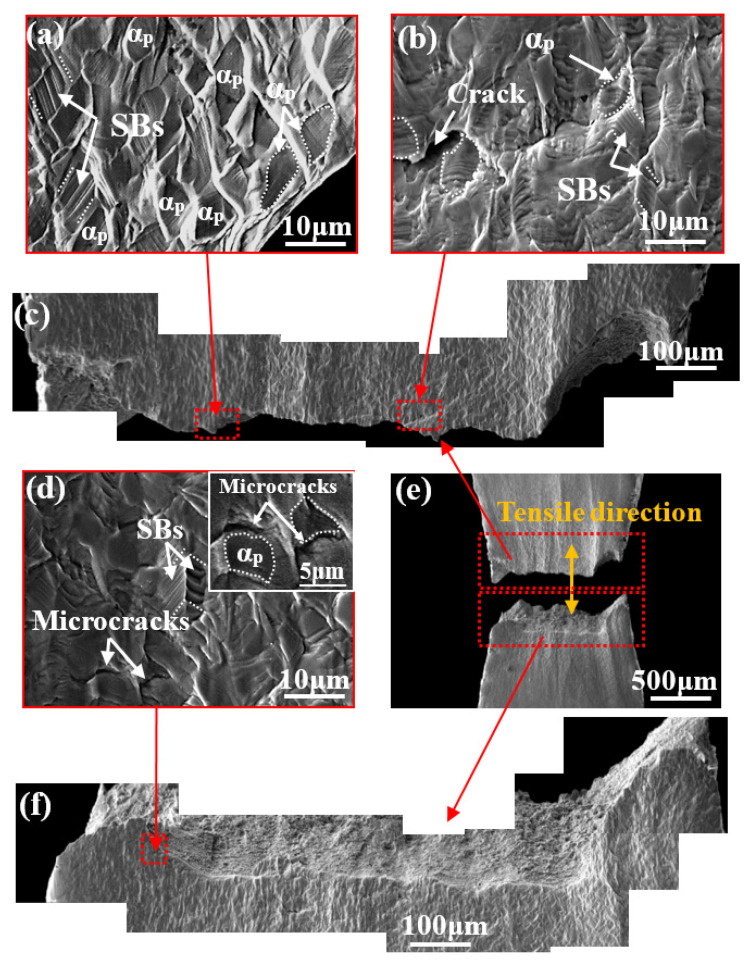
(**a**) The streamlined shape of the β matrix near the fractured zone at position C; (**b**) a crack propagated along the α_p_/β interface; (**c**,**f**) SEM images showing the rough fracture surface of the sample; (**d**) magnified image showing intensified SB formation in the α_p_ phase and microcracking at the α_p_/β interface; (**e**) macroscopical SEM images of the bimodal microstructure at position C.

Figure 7 illustrates in situ SEM images of the bimodal microstructure at position C (after fracture). The fracture surface is relatively rough (Figure 7e) and significant necking can be observed at the region close to the fracture surface (Figure 7c,f). As shown in Figure 7b,d, many microcracks form in the region close to the fracture surface: these nucleate at and generally propagate along the α_p_/β interface (the crack path is tortuous). Although the number of SBs increases, the α_p_ phase still maintains a granular shape (Figure 7d) and grains are wrapped by the significantly distorted β matrix (Figure 7d). As can be seen in Figure 7a, the morphology of the β matrix near the fractured zone becomes streamlined in shape, implying that it undergoes significant plastic deformation before fracture. Therefore, the β matrix undergoes greater deformation than the α_p_ phase during in situ stretching; in addition, this deformation is relatively uniformly distributed in the β matrix, probably due to the excellent deformation compatibility.

**Figure 8:**
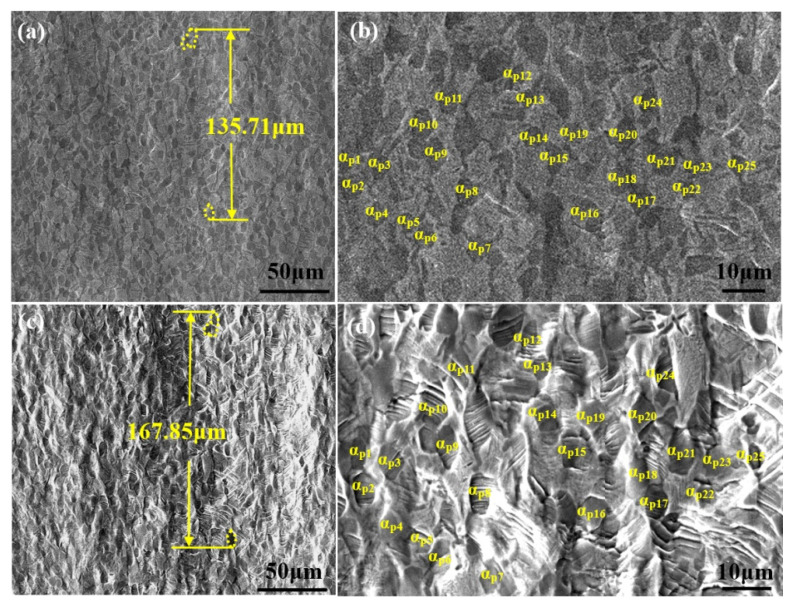
In situ SEM images of bimodal samples; (**a**) a selected area at position A; (**b**) magnified image showing the morphologies of α_p_ phase at position A; (**c**) the area in (**a**) stretched to position B; (**d**) magnified image illustrating the deformation within α_p_ phase at position B.

To assess the deformation behavior of the bimodal microstructure during in situ tensile loading, an area of the sample was selected for tracking, and the SEM images thereof at different stages were recorded. Figure 8a,c show the SEM images of the same area on a specimen with a bimodal microstructure surface at the stage of positions A and B, respectively: with increasing strain, the sample surface became significantly rougher and took on an undulate appearance (Figure 8c). To determine the strain in this local area, the changes in distance between two α_p_ phase grains on the sample surface were measured. Stretched from position A to position B, the distance increased from 135.71 μm (position A) to 167.85 μm (position B), indicating some 23.68% of the plastic deformation appears along the tensile direction (*ε_L_*) in this region.

Figure 8b,d are magnified images of Figure 8a,c, respectively. Although the sample underwent severe plastic deformation, the distribution of SBs in the β matrix remained relatively uniform. For further analysis, 25 α_p_ phase grains were selected for calibration (and assigned serial numbers 1 to 25). The size changes of these 25 α_p_ phase grains along the tensile direction from positions A to B were statistically studied (Table 1); most α_p_ phase regions have *ε_L_* values of less than 10%, and their average *ε_L_* value is 9.72%, which is only 41% of the average *ε_L_* value over the region. This result demonstrates that during tensile deformation, the β matrix was subject to larger plastic deformation than the α_p_ phase.

#### 3.3.2. Evolution of the Lamellar Microstructure

**Figure 9:**
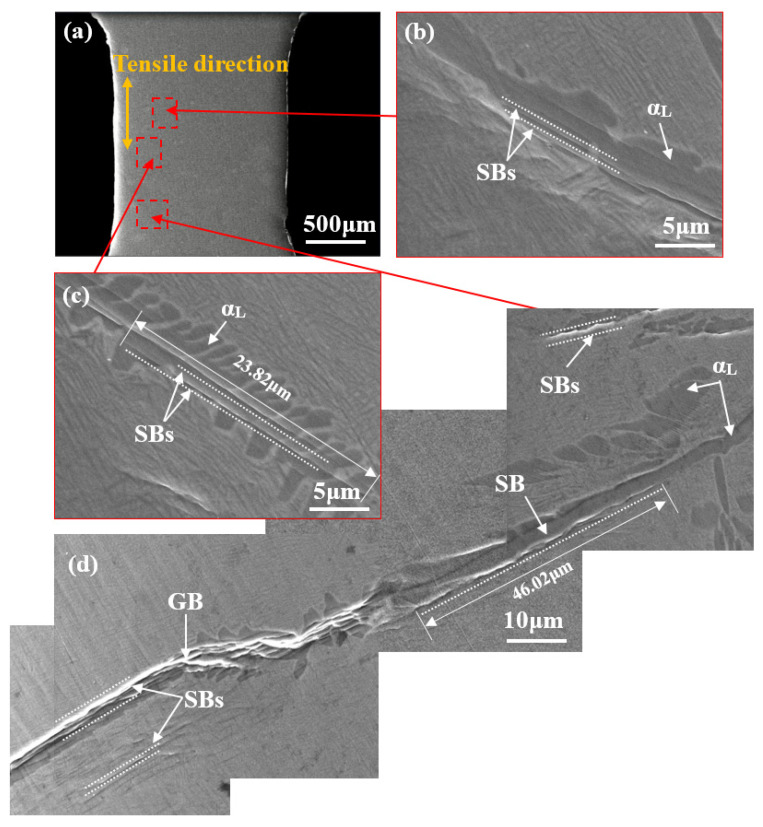
(**a**) In situ SEM images of lamellar microstructure at position A’; (**b**,**c**) magnified image showing several SBs in an α_L_ phase or at its interface; (**d**) magnified image showing plentiful SBs in a long coarse α_L_ phase at a GB at an angle of about 45° to the tensile direction.

Figure 9 shows BSE-SEM images of specimens with a lamellar microstructure during in situ stretching at position A’ on the stress–displacement curve (Figure 4). As shown in Figure 9d, some parallel SBs form within or at the boundaries of the α_L_ phase at an angle of about 45° to the tensile direction. Differing from those in specimens with a bimodal microstructure, in which such SBs measure only several microns, these SBs aligned along the length of the α_L_ phase are significantly longer and can always grow to several tens of microns.

**Figure 10:**
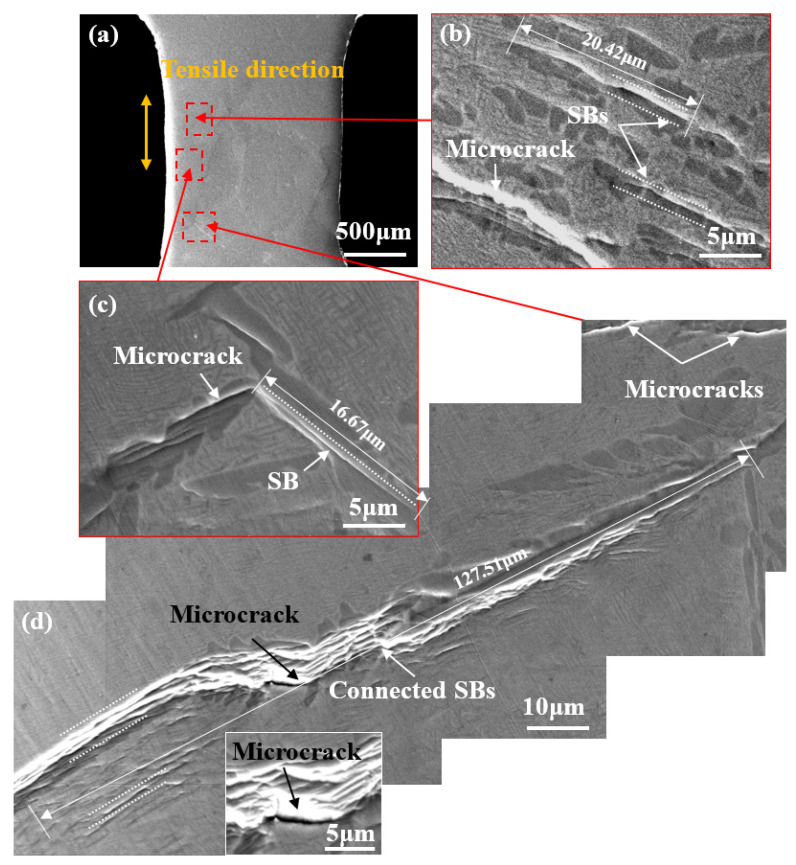
(**a**) In situ SEM images of lamellar microstructure at position B’; (**b**,**c**) magnified image showing several microcracks formed in α_L_ phase or at their interfaces; (**d**) magnified image showing SBs connected with each other and forming an SB with a length of 127.51 μm.

Figure 10 illustrates BSE-SEM images of lamellar samples during in situ stretching at position B’. As the strain increases, SBs gradually extend and connect with each other along the length of α_L_ at which some extremely long SBs are formed. As shown in Figure 10d, the connected SBs in the α_L_ at the grain boundary are over one hundred microns in length, besides which certain microcracks evolving from SBs can also be found in the α_L_ grains or at their interfaces.

**Figure 11:**
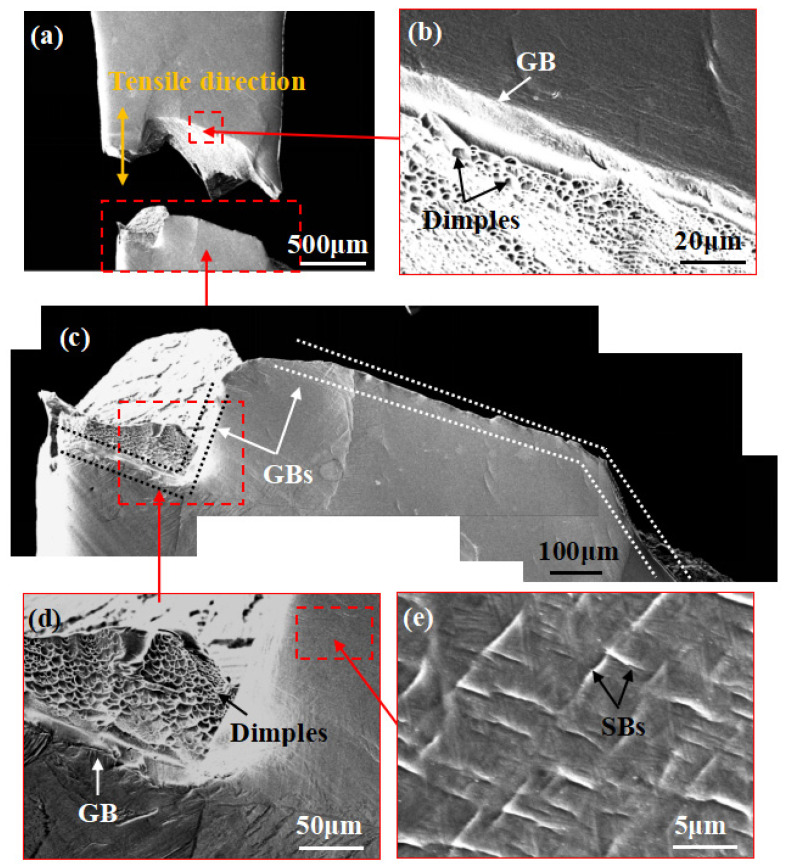
(**a**) In situ SEM images of the lamellar microstructure at position C’; (**b**,**d**) magnified images showing many small, shallow dimples on the fracture surface; (**c**) magnified image showing the sample with a shape fracture surface generally breaking along the GB; (**e**) some short crossed SBs are shown in the β matrix adjacent to the fracture surface.

Figure 11 exhibits in situ SEM images of lamellar samples during stretching at position C’. No obvious necking occurs in the sample until it fractures, which indicates that the lamellar microstructure undergoes less plastic deformation than the bimodal microstructure. Although there are slight SBs in the β matrix adjacent to the fracture surface, they are fewer in number than in specimens with a bimodal microstructure. Judging from Figure 11c,d, specimens with a lamellar microstructure generally fracture along the GBs, which results in a sharp fracture surface.

**Figure 12:**
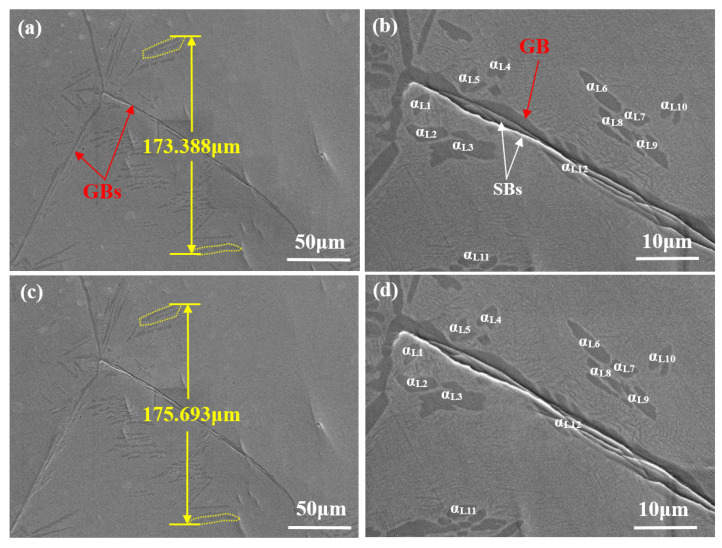
In situ SEM images of the lamellar microstructure; (**a**) a selected area at position A’; (**b**) magnified image showing the morphologies of α_L_ phase at position A’; (**c**) the area in (**a**) stretched to position B’; (**d**) magnified image illustrating the deformation within α_L_ phase at position B’.

As shown in Figure 12a,c, the changes in a selected area of the lamellar microstructure from position A’ to position B’ are tracked. The changes in roughness of the sample surface are small and there are fewer SBs compared with those in specimens with a bimodal microstructure (Figure 11). The ε_L_ value in this local area from position A’ to position B’ is found to be 1.33% (less than that in specimens with a bimodal microstructure).

Figure 12b,d demonstrate magnified versions of Figure 12a,c, respectively. To quantify the deformation behavior, 12 α_L_ phase grains were selected for calibration and assigned serial numbers 1 to 12. As shown in Figure 12b, SBs are mainly found in the large α_L_ phase (α_L12_), which can extend along the GB, while they are scarcely found in the β matrix or small α_L_ phase regions. With the increase in strain from position A’ to position B’, SBs in α_L12_ deepen, while the microstructure in the β matrix and small α_L_ phase changes little (Figure 12d). The ε_L_ values of the selected 12 α_L_ phase grains deformed from position A’ to position B’ were statistically analyzed (Table 2). According to the data in Table 2, the relatively small α_L_ phase (α_L1_-α_L11_) has quite small ε_L_ values (less than 1.0%), while the ε_L_ value of the long α_L_ phase at the GB (α_L12_) is found to be 5.79%, which is 3.35 times greater than the ε_L_ value across this area; the deformation in the lamellar microstructure is mainly concentrated in the large α_L_ phase found at the GB (i.e., α_L12_).

## 4. Discussion

Recently, investigators have found that mechanical behavior and its related mechanism of action are sensitive to the initial microstructure of titanium alloys. Specimens with a bimodal microstructure are found to have a lower strength but higher ductility than those with a lamellar microstructure [11,12,13,14,15], while the essential reason for the differences in mechanical behavior between bimodal and lamellar microstructures is still hotly debated. Huang et al. [23] found that the α_p_ phase in specimens with a bimodal microstructure plays a major role in accommodating the plastic strain of titanium alloys due to its good compatibility during deformation, while Tan et al. [24] stated that cracks are readily initiated at SBs in the α_p_ phase, which lies at the crux of the tensile deformation. Liu et al. [25,26] considered that a high stress concentration at the α_L_/β grain boundary results in intergranular fracture and low ductility of specimens with a lamellar microstructure, whereas Qin et al. [27] found that the crack nucleates inside the β grains and will spread under high tensile stress without hindrance in the larger β grains, leading to the low plasticity of specimens with a lamellar microstructure. In this study, their quite different mechanical properties were found to be essentially attributed to different microstructural evolutions during tensile loading.

### 4.1. Deformation Mechanisms and Microstructural Evolution of the Bimodal Microstructure

**Figure 13:**
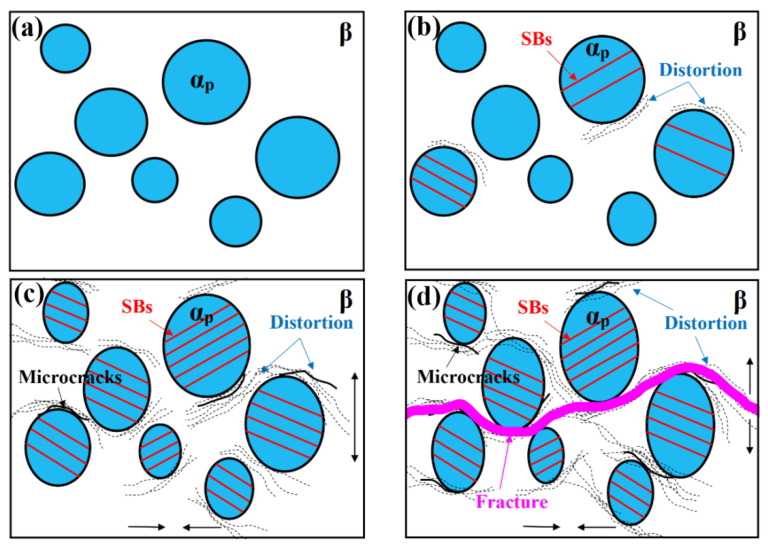
Schematic mechanism showing the evolution of bimodal microstructure during in situ stretching; (**a**) the initial microstructure; (**b**) at a relatively low strain; (**c**) at a relatively high strain; (**d**) schematic mechanism of deformation of bimodal samples after fracture.

Based on the results of in situ SEM observation, the deformation mechanisms and microstructural evolution of the bimodal microstructure are shown schematically in Figure 13.

**Figure 14:**
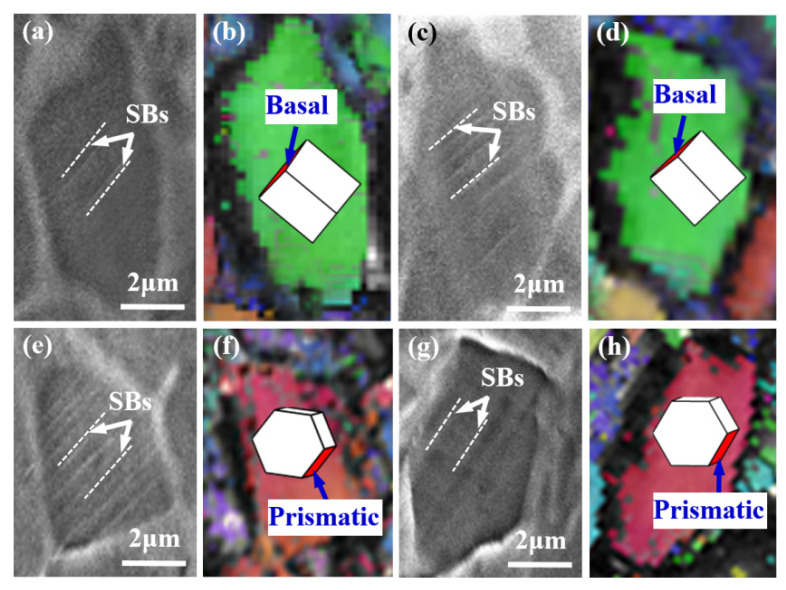
Examples of identification of activated slip systems on the α_p_ phase in bimodal microstructure; (**a**,**b**) a basal slip activates (SF = 0.38) in the α_p_ phase; (**c**,**d**) a basal slip activates (SF = 0.37) in the α_p_ phase; (**e**,**f**) a prismatic slip activates (SF = 0.45) in the α_p_ phase; (**g**,**h**) a prismatic slip activates (SF = 0.46) in the α_p_ phase.

The bimodal microstructure contains coarse globular α_p_ grains distributed in the β matrix (Figure 13a). Under a relatively small strain (Figure 13b), many parallel, deep SBs are formed inside some α_p_ phase regions due to the limited slip systems of α_p_ and the stress concentration caused by their relatively large size. As previously proved by Semiatin et al. [28], the ratio of critical resolved shear stress in the α phase at room temperature was 1:0.7:3.2 for basal ({0001}<11−20>), prismatic ({1−100}<11−20>), and pyramidal slip ({1−101}<11−20>), respectively. This indicates that pyramidal slip is difficult to take place and the α_p_ phase is more likely to slip along its basal or prismatic plane at room temperature. Figure 14 demonstrates four examples of SB identification for a bimodal microstructure after in situ stretching. These SBs essentially correspond to prismatic or basal slip systems and to single slip behavior with a relatively large Schmid factor (SF) (SF > 3.7), which agrees well with the results of Semiatin et al. [28].

**Figure 15:**
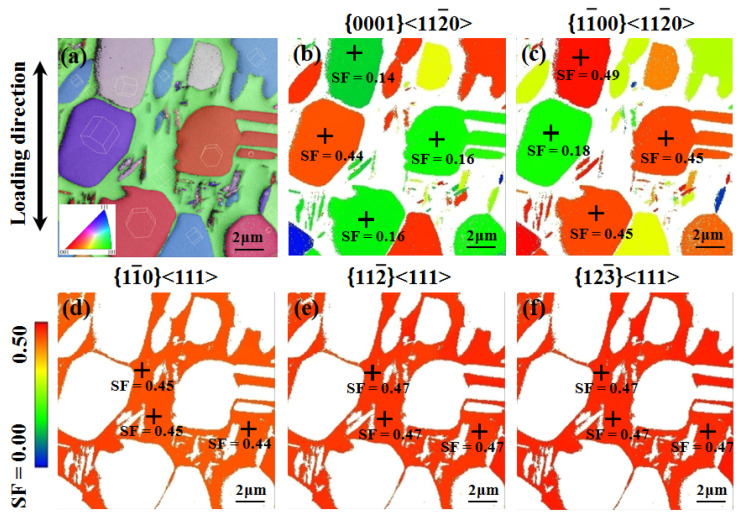
EBSD images of bimodal microstructure before stretching in situ; (**a**) inverse pole-figure (IPF) map; (**b**) SF map of α for basal slip; (**c**) SF map of α for prismatic slip; (**d**) SF map of β for {1−10}<111> slip; (**e**) SF map of β for {11−2}<111> slip; (**f**) SF map of β for {12−3}<111> slip.

In addition, as shown in Figure 15a, the orientations of α_p_ phases are randomly distributed in the bimodal microstructure. For the same α_p_ phase, there is a large difference in SF values for basal and prismatic slips (Figure 15b,c); the one with the maximum SF is supposed to be the easiest to activate [21]. Therefore, it is difficult for the α_p_ phase to activate both basal and prismatic slips simultaneously. SBs are prone to occur along a single basal or prismatic plane of the α_p_ phase, which is oriented solely along the maximum shear stress direction, i.e., at an angle of 45° to the applied tension. Differing from the α_p_ phase, there are significantly more slip systems in the β phase, among which {1−10}<111>, {11−2}<111>, and {12−3}<111> slips were thought to be the three typical cases [28,29]. As presented in Figure 15d,f, there is no significant difference in the SF values for these three types of slips in the β phase. Thus, it is difficult to observe β phase regions’ slip strictly along one single plane especially under a small strain.

In the bimodal microstructure, although a small number of SBs in the α_p_ phase can pass through the α_p_/β interface, most are restricted at the interface between β and α_p_ and confined to within a single α_p_ phase grain. This will lead to an increased stress concentration at the α_p_/β interface, therefore distorting the interface. As the strain increases (Figure 13c), the α_p_ phase is elongated slightly along the tensile direction (Figure 8).

β is softer than α_p_ due to lower concentrations of solute Al in β [30], thus β should bear more significant deformation than the α_p_ phase at the same stress, while due to the more numerous slip systems and greater deformation coordination in BCC β, parallel, deep SBs may be less likely to form in the β phase when the strain is relatively small (Figure 6 and Figure 7). Caused by the different deformation behaviors between α_p_ and β, stress concentration at the interface gradually increases with increasing strain, finally generating microcracks. Then, with the further increase in strain (Figure 13d), the number of SBs in the α_p_ phase increases slightly due to its more limited slip system, while that in the β matrix increases to a much greater extent. This further aggravates the stress concentration at the α_p_/β interface, making the microcrack propagate along the α_p_/β interface and into the β matrix. As the microcracks grow, they gradually come closer together, whereupon they tend to interconnect to form a main crack. Additionally, as the main crack grows, it is likely to bridge these microcracks formed at the α_p_/β interfaces. This finally leads to the zig-zag crack path and rough fracture surface of the sample since the α_p_ grains are randomly distributed in the β matrix (Figure 13d). 

It should also be pointed out that, because of the connected distribution of the β phase and isolated distribution of the α_p_ phase in specimens with a bimodal microstructure, localized deformation readily propagates into the surrounding area by way of the soft β phase, with only a minor role played by the harder α_p_ phase. This ensures relatively uniform deformation, giving rise to the excellent ductility of the bimodal microstructure.

### 4.2. Deformation Mechanisms and Microstructural Evolution of the Lamellar Microstructure

**Figure 16:**
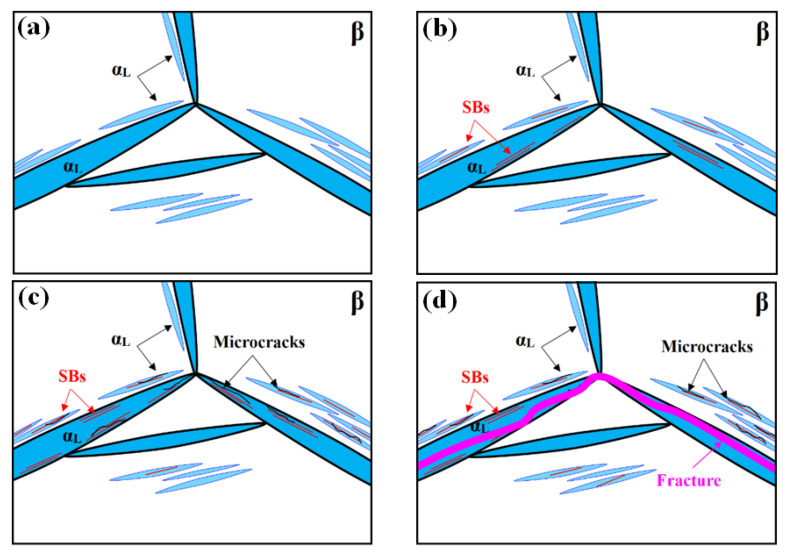
Schematic mechanism showing the evolution of lamellar samples during in situ stretching; (**a**) initial microstructure; (**b**) at a relatively low tensile displacement; (**c**) at a relatively high tensile displacement; (**d**) schematic mechanism of the deformation of lamellar samples after fracture.

Figure 16 schematically presents the deformation mechanisms and microstructural evolution of the lamellar microstructure based on the results of in situ SEM observation.

**Figure 17:**
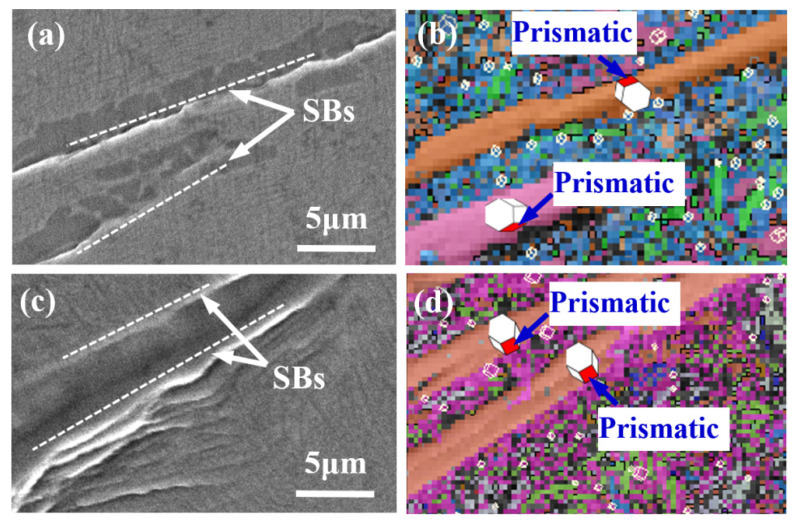
Examples of identification of activated slip systems on α_L_ phase in lamellar microstructure; (**a**,**b**) prismatic slips activate in two α_L_ phase with SF values of 0.44 and 0.42, respectively; (**c**,**d**) prismatic slips activate in two α_L_ phases with SF values of 0.43 and 0.45, respectively.

Differing from the bimodal microstructure, many long and coarse lamellar α_L_ grains, rather than globular α_p_ regions, are seen in the lamellar microstructure: these are mainly distributed at or near GBs (Figure 16a). Since these α_L_ phase regions are much harder than the β phase [31], they can exert a strong fencing effect and therefore separate each β grain into a relatively isolated region between which dislocation cannot easily pass. This in turn leads to a great stress concentration at the α_L_ phase. Therefore, deep SBs are first observed in the coarse α_L_ phase at GBs under a relatively low tensile strain (Figure 16b). As shown in Figure 17, these SBs correspond to prismatic slip behavior (SF > 4.2) and generally lie along the plane at an angle of approximately 45° to the direction of the applied tension (i.e., the direction of maximum shear stress).

Meanwhile, the deformation in the β matrix is much smaller due to the strong fencing effect of the α_L_ (Figure 9). With increasing strain, the number of SBs in the large α_L_ phase increases while that in the β matrix remains low (Figure 10), suggesting that the deformation in lamellar microstructures is non-uniform and mainly concentrated in the large α_L_ grains at GBs. As a result of this inhomogeneous deformation, microcracks are readily initiated from, and propagate along, the SBs (Figure 16c). The formation of microcracks in these α_L_ regions in turn produces a greater stress concentration, releasing stress accumulation in the β matrix. This aggravates the inhomogeneity of the deformation of specimens with a lamellar microstructure. Finally, the sample fractures along the large α_L_ grains at GBs, leading to the low plasticity of the lamellar microstructure (Figure 16d).

## 5. Conclusions

In this study, the microstructural evolution and fracture mechanisms of a Ti–5Al–5Mo–5V–1Cr–1Fe alloy with bimodal and lamellar microstructures were investigated through in situ tensile SEM observation. The following conclusions can be obtained:

1. For the bimodal microstructure, parallel and deep SBs, at around 45° to the tensile direction, are first observed in the α_p_ phase due to the limited slip systems therein and the stress concentration caused by its large size. These SBs mainly correspond to prismatic or basal slip systems. With increasing strain, distortion at the α_p_/β interface arises, leading to the evolution of microcracks: the interconnection of the microcracks at the α_p_/β interface finally leads to the fracturing of the bimodal microstructure.
2. For the lamellar microstructure, parallel and deep SBs are first observed in coarse α_L_ phase grains or its interfaces at GBs, making an angle of around 45° to the tensile direction. With increasing strain, these SBs grow along the length of the α_L_ phase and gradually interconnect, thus forming microcracks. The lamellar microstructure finally fractures along the α_L_ phase at GBs through the interconnection of those microcracks in the α_L_ phase.
3. Due to the connected distribution of β and isolated distribution of the α_p_ phase in the bimodal microstructure, the localized deformation readily propagates into the surrounding area through β, which is softer and has a better ability to undergo plastic deformation. This results in the more uniform deformation and higher ductility of specimens with a bimodal microstructure.
4. The coarse α_L_ phase at the GBs greatly restricts the deformation in the β matrix during tensile loading, which in turn produces a significant stress concentration and local deformation in the coarse α_L_ phase. This finally leads to intergranular fracture and contributes to the higher strength and lower ductility of those specimens with a lamellar microstructure.

## Figures and Tables

**Table 1:** Calculation of *ε_L_* values for αp phase (Figure 8) from positions A to B.

No.	Position A (μm)	Position B (μm)	Δ (Position A→B) (μm)	*ε_L_* (%)
α_p1_	6.54	7.15	0.61	9.33
α_p2_	6.78	7.30	0.52	7.67
α_p3_	6.84	7.52	0.68	9.94
α_p4_	5.67	6.13	0.46	8.11
α_p5_	5.34	5.77	0.43	8.05
α_p6_	6.78	7.44	0.66	9.73
α_p7_	7.12	7.73	0.61	8.57
α_p8_	7.46	8.32	0.86	11.53
α_p9_	8.64	9.43	0.79	9.14
α_p10_	7.54	8.61	1.07	14.19
α_p11_	8.13	9.27	1.14	14.02
α_p12_	5.49	5.98	0.49	8.93
α_p13_	4.91	5.39	0.48	9.78
α_p14_	5.17	5.62	0.45	8.70
α_p15_	7.80	8.25	0.45	5.77
α_p16_	6.19	6.86	0.67	10.82
α_p17_	6.89	7.29	0.40	5.81
α_p18_	8.22	9.12	0.90	10.95
α_p19_	5.94	6.43	0.49	8.25
α_p20_	9.83	10.57	0.74	7.53
α_p21_	8.14	9.36	1.22	14.99
α_p22_	4.87	5.21	0.34	6.98
α_p23_	5.59	6.28	0.69	12.34
α_p24_	8.64	9.78	1.14	13.19
α_p25_	6.19	6.72	0.53	8.56
			Average	9.72

**Table 2:** Calculation of *ε_L_* values of α_L_ phase (Figure 12) from positions A’ to B’.

No.	Position A’ (μm)	Position B’ (μm)	Δ (Position A’→B’) (μm)	*ε_L_* (%)
α_L1_	3.441	3.459	0.018	0.52
α_L2_	2.957	2.973	0.016	0.54
α_L3_	6.068	6.108	0.040	0.66
α_L4_	3.925	3.934	0.009	0.23
α_L5_	4.084	4.108	0.024	0.59
α_L6_	7.189	7.257	0.068	0.95
α_L7_	2.996	3.012	0.016	0.53
α_L8_	5.839	5.874	0.035	0.60
α_L9_	6.226	6.283	0.057	0.92
α_L10_	5.144	5.181	0.037	0.72
α_L11_	2.527	2.545	0.018	0.71
α_L12_	26.774	28.324	1.550	5.79
-	-	-	Average	1.06

## Data Availability

The data presented in this study are available on request from the corresponding author.
